# Therapeutic Role of Protein Tyrosine Phosphatase 1B in Parkinson's Disease via Antineuroinflammation and Neuroprotection *In Vitro* and *In Vivo*

**DOI:** 10.1155/2020/8814236

**Published:** 2020-12-29

**Authors:** Chien-Wei Feng, Nan-Fu Chen, Te-Fu Chan, Wu-Fu Chen

**Affiliations:** ^1^Department of Obstetrics and Gynecology, Kaohsiung Medical University Hospital, Kaohsiung 807377, Taiwan; ^2^Center for Cancer Research, Kaohsiung Medical University, Kaohsiung 807377, Taiwan; ^3^Division of Neurosurgery, Department of Surgery, Kaohsiung Armed Forces General Hospital, Kaohsiung, Taiwan; ^4^Department of Neurosurgery, Kaohsiung Chang Gung Memorial Hospital and Chang Gung University College of Medicine, Kaohsiung, Taiwan; ^5^Department of Neurosurgery, Xiamen Chang Gung Hospital, Xiamen, Fujian, China; ^6^Department of Marine Biotechnology and Resources, National Sun Yat-sen University, Kaohsiung, Taiwan

## Abstract

Parkinson's disease (PD) is one of the most widespread neurodegenerative diseases. However, the currently available treatments could only relieve symptoms. Novel therapeutic targets are urgently needed. Several previous studies mentioned that protein tyrosine phosphatase 1B (PTP1B) acted as a negative regulator of the insulin signal pathway and played a significant role in the inflammation process. However, few studies have investigated the role of PTP1B in the central nervous system. Our study showed that suramin, an inhibitor of PTP1B, could improve neuronal damage. It could significantly attenuate the interferon-gamma-induced upregulation of proinflammatory cytokines, including inducible nitric oxide synthase (iNOS), cyclooxygenase-2 (COX-2), and nuclear factor kappa-light-chain-enhancer of activated B cells (NF-*κ*B). It enhanced M2 type microglia markers, such as arginase-1 and Ym-1 in BV2 murine microglial cells. PTP1B inhibition also reversed 6-hydroxydopamine- (6-OHDA-) induced downregulation of phospho-cAMP response element-binding protein (p-CREB) and brain-derived neurotrophic factor (BDNF) in SH-SY5Y cells. Besides, we knocked down and overexpressed PTP1B in the SH-SY5Y cells to confirm its role in neuroprotection. We also verified the effect of suramin in the zebrafish PD model. Treatment with suramin could significantly reverse 6-OHDA-induced locomotor deficits and improved tyrosine hydroxylase (TH) via attenuating endoplasmic reticulum (ER) stress biomarkers. These results support that PTP1B could potentially regulate PD via antineuroinflammation and antiapoptotic pathways.

## 1. Introduction

Over the last few decades, the NIH reported that approximately 50,000 patients were diagnosed with Parkinson's disease (PD) every year in America. Moreover, the global PD pharmaceutical market was estimated at 4.02 billion in 2016 which will increase to 6.48 billion USD in 2024 with a compound annual growth rate (CAGR) of 6.15%. About 1 million patients suffer from PD in the US [1, 2]. However, the current PD treatments can only relieve the clinical symptoms but cannot delay disease progression. No cure is currently available, and there is a tremendous demand for effective agents or strategies for the prevention or attenuation of PD progression. Several therapeutic targets have been investigated to help PD patients.

Protein tyrosine phosphatase 1B (PTP1B) is a member of the PTP family and widely expressed in almost every body tissue. Previous research showed that PTP1B is involved in modulating inflammation and participates in the cascade of the brain-derived neurotrophic factor (BDNF) pathway [3]. PTP1B is thought to be a negative regulator of several receptors and receptor-associated tyrosine kinases [4–6]. This protein dephosphorylates and inactivates several membrane receptor tyrosine kinases, such as epidermal growth factor receptor (EGFR) [7], platelet-derived growth factor receptor [5], insulin receptor [8], and insulin-like growth factor-1 receptor [9]. Hence, the dysfunction of PTP1B activity has proved to be involved in the pathogenesis of several human diseases, such as cancer, diabetes, obesity, and immune disorders [10, 11]. Thus, the research mentioned above used PTP1B as a therapeutic target because of its important role in diverse pathological conditions. However, very few studies have mentioned the role of PTP1B in neurological diseases. De Jonghe et al. reported that the inhibition of PTP1B led to enhanced thermoregulatory activity in brown adipose tissue and cold-induced attenuated physical activity in POMC neurons [12]. Besides, Ozek et al. showed that PTP1B overexpression could inhibit BDNF/Trk cascade and PTP1B inhibition prompted BDNF to combine more readily with the Trk receptor [3]. Then, Zhu et al. demonstrated that PTP1B was significantly elevated in rat spinal cord injury (SCI). They also suggested that the elevation of PTP1B expression probably regulated endoplasmic reticulum (ER) stress-induced neuronal apoptosis [13]. In addition to inhibiting BDNF/Trk signaling, PTP1B is involved in regulating immune cell signaling, which plays a key role in inflammation [14, 15].

The multifactorial PTP1B connection on PD-related pathways may provide more opportunities to slow PD progression. The dephosphorylation of JAK2, STAT6, or STAT3 inactivates the pathways that were induced by IL-4 [16, 17]. Previous studies showed that IL-4 plays an important role in the alternative activation of M2 macrophages. The biomarkers of M2 macrophages include the upregulation of the mannose receptor and arginase-1 (arg-1) protein expression which possesses anti-inflammatory activity [18, 19]. Previous studies showed that the activation of PTP1B reduces the ratio of M2 macrophages in total macrophages. Previous research has also indicated that mice with the myeloid-specific deletion of PTP1B could help protect mice against LPS-induced endotoxic shock and high-fat diet-induced inflammation [20]. Besides, an increase of systemic IL-10 levels with a corresponding decrease in IL-6 was also observed [20]. Traves et al. also revealed that PTP1B knockdown could not only attenuate the M1 downstream response but also enhance the activation of the M2 reaction in macrophages [21]. Because of PTP1B's role in inflammation, we attempted to investigate its implications in PD. Some studies have indicated that neuroinflammation may play a critical role in PD progression, including inflammatory cytokines [22, 23]. Some inflammatory cytokines, such as iNOS and COX-2, could represent the activity of M1 macrophage [24]. In addition, cytokines are upregulated via the translocation of nuclear factor kappa-light-chain-enhancer of activated B cells (NF-*κ*B). Some studies also indicated that the regulation of NF-*κ*B could provide greater neuroprotective benefits in PD treatment than conventional treatments [25–28]. However, no study investigated the role of PTP1B in neuroinflammation which affects PD progression. Apart from neuroinflammation, PTP1B also has an essential part in ER stress [29].

The continuous ER stress response contributes to apoptosis, inflammation, and lipid accumulation [30]. In PD, ER stress would cause the accumulation and deposits of misfolded proteins such as *α*-synuclein, which affects various cell signaling systems, defects in neuronal connectivity, and cell death [31–33]. Moreover, a mutation in the proteasome E3 ligase protein, called Parkin, also causes ER stress with the accumulation of cytotoxic fibrils and protein aggregates in cells [34]. Thus, some studies indicated that the inhibition of ER stress could probably slow PD progression [35–37]. The role of PTP1B in ER stress was first discovered by Gu et al. The study demonstrated that PTP1B modulates IRE-1-mediated ER stress signaling pathways. The research showed that a deficiency of PTP1B triggered the subsequent ER stress-related cascades, including impairment in XBP-1 splicing and ER degradation-enhancing *α*-mannosidase-like protein transcription. The phenomena mentioned above were inhibited in PTP1B-deficient embryonic mouse primary fibroblasts [38–40]. However, these studies focused on the peripheral condition. Thus, our study intended to explore PTP1B's role in the central nervous system, especially in PD. Our study takes advantage of suramin as an inhibitor of PTP1B. This compound was initially used as an antitrypanosomal drug, and some studies used it to inhibit PTP1B's activity. Zhang et al. (1998) first demonstrated that suramin could antagonize its active site to inhibit PTPase activity. We intend to use suramin to inhibit the activity of PTP1B in our study [41].

## 2. Materials and Methods

### 2.1. Cell Culture

The SH-SY5Y neuroblastoma cells were purchased from ATCC (Rockville, MD, USA) and incubated with 10% fetal bovine serum (FBS) in DMEM (Invitrogen/Gibco). BV2 murine microglial cells were incubated with 10% FBS in 1 : 1 DMEM and F12 medium. Both cell lines were maintained in an incubator with 5% CO_2_. The compound suramin was dissolved in dimethyl sulfoxide. For the cell survival assay, the cells were seeded in 96-well microplates (Corning, NY, USA) at an initial density of 3 × 10^4^ cells/well. The relative protection was calculated as 100 × (OD of 6-hydroxydopamine (6-OHDA) plus sample − OD of 6-OHDA)/(OD of control − OD of 6-OHDA) from Lee et al. The relative rates of neuroprotection of the control and 6-OHDA-treated alone groups were taken to be 100% and 0%, respectively. The neuroprotective activity method used was modified from previous studies [42, 43]. For the terminal deoxynucleotidyl transferase dUTP nick end labeling (TUNEL) analysis, SH-SY5Y cells were seeded at a density of 1 × 10^6^ cells/dish in 6 cm dishes. For western blotting, SH-SY5Y cells were grown on a 6 cm dish at a density of 1 × 10^6^ cells/dish.

### 2.2. Transfection of PTP1B siRNA in SH-SY5Y

To inhibit PTP1B expression in SH-SY5Y cells, these were transiently transfected with siRNAs (Santa Cruz Biotechnology, CO). The transient transfections with siRNAs were performed with LipofectAMINE PlusReagent (Invitrogen). After 3 h of transfection, the cultures were washed once and incubated with DMEM overnight. The next day, the cells were rinsed with fresh medium before being treated with other agents. Then, the cells were harvested for western blotting.

### 2.3. Transfection of PTP1B Plasmid in SH-SY5Y

When the cells had grown to approximately 50% confluence, the PTP1B (NM_002827, OriGene, USA) human PTPN1 (GFP-tagged) plasmid and LipofectAMINE PlusReagent (Invitrogen) were added to SH-SY5Y cells after changing the cell culture medium to DMEM without penicillin and streptomycin. After transfecting the cells with PTP1B plasmid for 24 h, DMEM supplemented with 10% FBS replaced the cell culture medium. GFP fluorescence was detectable after the cells were transfected with a plasmid for 48 h and the cells were selected. After one week of the selection, the cells were cultured for passage, and then PTP1B gene expression on the transcription and translation was assessed. Successfully transfected SH-SY5Y cells were named PTP1B-overexpressed SH-SY5Y cells.

### 2.4. TUNEL Assay

The *In Situ* Cell Death Detection Kit, POD (TUNEL assay, Roche Diagnostics GmbH, Mannheim, Germany), was used to assess apoptotic cells. SH-SY5Y cells were grown on coverslips. They were treated with 10 *µ*M of suramin for 1 h before a 20 *µ*M 6-OHDA incubation for 8 h. Cells were fixed for 1 h with a 4% paraformaldehyde solution after washing the cells thrice with PBS. Then, we blocked the cells with a 3% H_2_O_2_ solution in methanol for 10 min. The cells were incubated with a permeabilization solution for 2 min and washed thrice with PBS. Then, the TUNEL stain mixture was added, and the cells were incubated for 1 h at 37°C. Following this, 4′, 6-diamidino-2-phenylindole was added for 10 min, and the cells were washed thrice with PBS. Coverslips were mounted using glass slides and imaged using a Leica DM6000 microscope.

### 2.5. Zebrafish Maintenance

The AB strains of wild type zebrafish were used for this study. Embryos were collected after natural spawning, staged according to the standard criteria, and synchronously raised at 28.5°C in Hank's buffer (13.7 mM NaCl, 540 *μ*M KCl, 25 *μ*M Na_2_HPO_4_, 44 *μ*M KH_2_PO_4_, 300 *μ*M CaCl_2_, 100 *μ*M MgSO_4_, 420 *μ*M NaHCO_3_, and pH 7.4).

### 2.6. Locomotor Behavioral Test

Zebrafish larvae were treated with 250 *μ*M 6-OHDA (2-day after fertilization (dpf) to 4 dpf) in the absence or presence of different concentrations of suramin (0.1, 1, and 10 *μ*M) (9 hpf to 4 dpf) in a 24-well plate. At 5 dpf, the fish were transferred into 10 cm dishes (16 fish/dish), and swimming behavior was monitored by an animal behavior system with automated video tracking (Singa Technology Co.; catalog no. TM-01). The zebrafish behavior analysis process was used and described in our previous study [44].

### 2.7. Western Blotting

Western blotting in SH-SY5Y and zebrafish was performed as described in our previous study [45]. In brief, samples were mixed with equal volume of sample buffer (2% SDS, 10% glycerol, 0.1% bromophenol blue, 2% 2-mercaptoethanol, and 50-mM Tris-HCl (pH 7.2)). The mixtures were loaded onto 10% SDS-polyacrylamide gel and electrophoresed at 300 V for 60 min. After electrophoresis, the separated proteins were transferred onto a polyvinylidene difluoride membrane (Immobilon-P; pore size, 0.45 *µ*m; Millipore, MA, USA) at 300 mA for 90 min at 4°C in a transfer buffer (50 mM Tris-HCl, 380 mM glycine, 1% SDS, and 20% methanol). The membrane was blocked for 40 min at room temperature with 5% nonfat dry milk and 0.1% Tween 20 in 20 mM Tris-HCl and 137 mM NaCl (pH 7.4) (TTBS) and incubated overnight at 4°C in the primary antibodies. The membrane was washed three times in TTBS for 10 min, blocked with 5% nonfat dry milk/TTBS, and then incubated for 1 h at room temperature with the secondary antibody, that is, horseradish peroxidase-conjugated anti-rabbit antiserum (dilution, 1 : 2500). Immunoblotting was performed using appropriate antibodies and horseradish peroxidase-conjugated secondary antibody. The immune reactive bands were captured by enhanced chemiluminescence (ECL kit; Millipore, Bedford, MA). The images were visualized using the UVP BioChemi Imaging System, and relative densitometric quantification was performed using LabWorks 4.0 software (UVP, Upland, CA). Monoclonal antibodies against *β*-actin (A-5441; Sigma) were used as the internal control for protein loading, and data were expressed as the ratio of the protein of interest to *β*-actin. The relative variations between the bands of the various treatment samples and the control group were calculated using the same image.

### 2.8. Quantitative Polymerase Chain Reaction

Zebrafish embryos at 9 hpf were treated for 87 h with 10 *μ*M suramin. Total RNA was extracted from 20 zebrafish larvae of each group using the TRIzol Reagent (Invitrogen™, USA). RNA was reverse transcribed to single-stranded cDNA using the iScript cDNA synthesis kit (Bio-Rad, Hercules, CA, USA). Reverse transcription polymerase chain reaction (RT-PCR) was performed using the gene expression assay primers for zebrafish as follows: PTP1B: F: 5′-CTTCACCGAGAGCATCACAA-3′ and R: 5′-GTTCGTCGGGTTGTTCATTT-3′; BDNF: F: 5′-ATAGTAACGAACAGGATGG-3′ and R: 5′-GCTCAGTCATGGGAGTCC-3′; iNOS: F: 5′-GGAGATGCAAGGTCAGCTTC-3′ and R: 5′-GGCAAAGCTCAGTGACTTCC-3′. We then performed real-time PCR using the iQ™ SYBR Green (Bio-Rad, Hercules, CA, USA) supermix for zebrafish in the Bio-Rad real-time PCR system (all materials were from Applied Biosystems). Each gene's expression level was presented as the relative fold change (log2 ratio), which was calculated using the comparative Ct method with GAPDH as the internal reference.

### 2.9. Chemicals and Antibodies

1. 6-OHDA (6-hydroxydopamine; Sigma, St. Louis, MO, USA; catalog: H4381)
2. *β*-Actin (loading control; Sigma, St. Louis, MO, USA; catalog: A5441)
3. Suramin (Sigma, St. Louis, MO, USA; catalog: S2671)
4. iNOS (BD Pharmingen, San Diego, CA, USA; catalog: 610600)
5. COX-2 (Cayman Chemical, Ann Arbor, MI, USA; catalog: 160126)
6. NF-*κ*B (Merck Millipore, Massachusetts, USA; catalog: MAB3026)
7. Ym-1 (Abcam, Biorbyt, Cambridge, UK; catalog: ab192029)
8. Arg-1 (Proteintech, Chicago, USA; catalog: 16001-1-AP)
9. PTP1B (Proteintech, Chicago, USA; catalog: 11334-1-AP)
10. Phospho-eukaryotic initiation factor 2 (p-eIF2) (antibodies-online; catalog: ABIN2776588)
11. Binding immunoglobulin protein (GRP-78) (Proteintech, Chicago, USA; catalog: 11587-1-AP)
12. Phospho-extracellular signal-regulated kinase (p-ERK) (Cell Signaling Technology, Danvers, MA, USA, Thr202/204; catalog: 9101)
13. Caspase-3 (IMGENEX, San Diego, CA, USA; catalog: Img-144A)
14. Tyrosine hydroxylase (Merck Millipore, Massachusetts, USA; catalog: MAB3018)

## 3. Results

### 3.1. Effects of Suramin on Interferon-Gamma- (IFN-*γ*-) Induced Upregulation of iNOS, COX-2, and IL-4-Induced Upregulation of Arg-1 and Ym-1 Protein Expression in BV2 Microglial Cells

**Figure 1:**
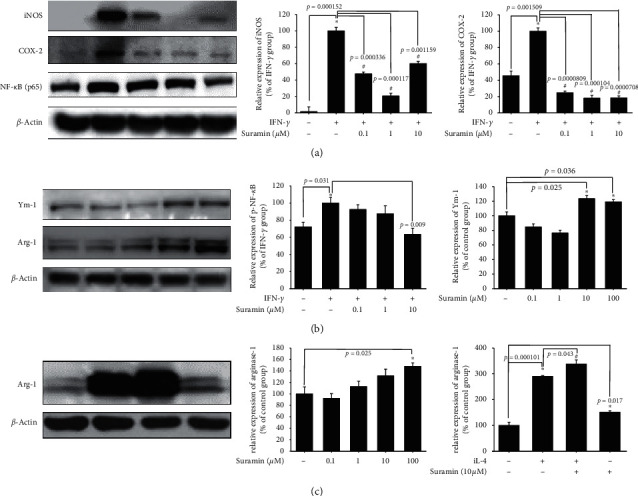
Effect of suramin on interferon-gamma- (IFN-*γ-*) induced upregulation of iNOS (inducible nitric oxide synthase) and COX-2 (cyclooxygenase-2) and increased Ym-1 and arginase-1 (arg-1) in BV2 murine microglial cells. (a) BV2 cells were cotreated with 0.1, 1, or 10 *μ*M suramin and challenged with 10 U/mL of IFN-*γ* for 24 h; western blotting for iNOS, COX-2, and NF-*κ*B (p65) of the control; IFN-*γ*; and IFN-*γγ* plus 0.1, 1, or 10 *μ*M suramin group is shown. *β*-Actin was used as an internal control. Data are presented as mean ± SEM, and each value contains three replicates and three samples. ^*∗*^Significantly different from the control group; ^#^significantly different from the IFN-*γ* group. (b) BV2 cells were treated with 0.1, 1, 10, and 100 *μ*M suramin for 24 h; western blotting for Ym-1 and arg-1 proteins of the control, 0.1, 1, 10, or 100 *μ*M suramin group is shown. *β*-Actin was used as an internal control. Data are presented as mean ± SEM, and each value contains three replicates and three samples. ^*∗*^Significantly different from the control group. (c) BV2 cells were cotreated with 10 *μ*M suramin and challenged with IL-4 for 24 h; western blotting for the arg-1 protein of the control; IL-4; and IL-4 plus 0.1, 1, 10, or 100 *μ*M suramin group is shown. *β*-Actin is used as an internal control. Data are presented as mean ± SEM, and each value contains three replicates and three samples. ^*∗*^Significantly different from the control group; ^#^significantly different from the IL-4 group.

We examined the anti-inflammatory activity in BV2 murine microglial cells using western blotting. The BV2 cells were pretreated with 0.1, 1, or 10 *μ*M suramin for 10 min and 10 U/mL of IFN-*γ* for 16 h. Our data demonstrated that IFN-*γ* significantly increased iNOS and COX-2 protein expression in BV2 murine microglial cells and 0.1, 1, and 10 *μ*M suramin significantly inhibited iNOS, COX-2, and NF-*κ*B (p65) protein expression (Figure 1(a)). Moreover, a dose-dependent curve was demonstrated for the administration of 0.1 and 1 *μ*M suramin. Then, the effect of PTP1B inhibition on alternative activation of microglia was checked. We examined M2 type microglia-related proteins, arg-1, and Ym-1. We showed that treatment with suramin alone (10 and 100 *μ*M) for 16 h could significantly enhance arg-1 and Ym-1 protein expression (Figure 1(b)). Our data showed that treatment with 20 ng/mL IL-4 for 16 h significantly increased arg-1 protein expression. We also observed that 10 *μ*M suramin significantly enhanced the IL-4-induced upregulation of arg-1 protein expression (Figure 1(c)). Moreover, the administration of 10 *μ*M suramin alone also showed a significant increase in arg-1 protein expression. These data indicate that the PTP1B inhibition may involve the regulation of polarization in microglial cells. Alternative activation was initiated. After confirmation of the anti-inflammatory effect, we then examined the antiapoptotic effect of PTP1B inhibition in neuron.

### 3.2. Effect of Suramin on 6-OHDA-Induced Apoptosis in SH-SY5Y Neuroblastoma Cell Line

**Figure 2:**
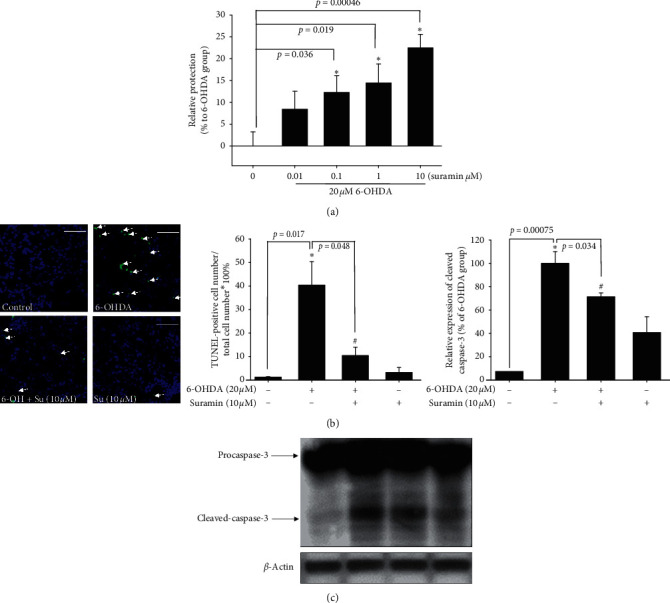
The neuroprotective effect of suramin on 6-OHDA-induced damage in SH-SY5Y cells. (a) SH-SY5Y cells were pretreated with 0.01, 0.1, 1, and 10 *μ*M suramin for 1 h and then challenged with 20 *μ*M 6-OHDA for 16 h in the control, 6-OHDA, 6-OHDA plus suramin, and suramin alone groups. The 6-OHDA-treated group was normalized as 0%. Data are presented as mean ± SEM, and each value contains three replicates and six samples. ^*∗*^Significantly different from the 6-OHDA group. (b) SH-SY5Y cells were pretreated with 10 *μ*M suramin and then challenged with 20 *μ*M 6-OHDA for 8 h in the control, 6-OHDA, 6-OHDA plus suramin, and suramin alone groups. TUNEL staining was performed, and white arrows represent apoptotic cells (scale bar, 100 *μ*M). The quantification of apoptotic cells is shown. (c) Western blotting of cleaved caspase-3 protein and quantification of each group. Data are presented as mean ± SEM, and each value contains three replicates and six samples. ^*∗*^Significantly different from the control group; ^#^significantly different from the 6-OHDA group.

We first checked the antiapoptotic effect of PTP1B inhibition by the inhibitor suramin on the SH-SY5Y neuroblastoma cell line. Apoptosis was validated with the following tests: relative protection, TUNEL staining, and cleavage caspase-3 protein expression. SH-SY5Y cells were pretreated with 0.01, 0.1, 1, and 10 *μΜ* suramin for 1 h, followed by 20 *μΜ* 6-OHDA treatment for 16 h. AlamarBlue was added to assess cell viability at 16 h. Our data revealed that 0.1, 1, and 10 *μΜ* suramin significantly reversed 6-OHDA-induced neuronal toxicity (Figure 2(a)). TUNEL was further used for detecting DNA fragmentation by labeling the 3′-hydroxyl termini on the double-stranded DNA breaks generated during apoptosis. Incubation with 20 *μ*M 6-OHDA for 8 h increased TUNEL staining compared with the control group, and the administration of 10 *μ*M suramin significantly reduced the number of TUNEL-positive cells (Figure 2(b)). The exposure of 6-OHDA increased the ratio of TUNEL-positive cells from 1.24% ± 0.20% to 40.35% ± 9.98%. Also, the pretreatment of 10 *μ*M suramin reduced the ratio from 40.35% ± 9.98% to 10.42% ± 3.54%. Then, we further checked the activated caspase-3 protein expression in 6-OHDA-induced SH-SY5Y cells. Our result demonstrated that 10 *μΜ* suramin significantly attenuated 6-OHDA-induced upregulation of cleaved caspase-3 protein expression (Figure 2(c)). Except for the antiapoptotic cascade, we also examined the neuroprotective pathways including phospho-cAMP response element-binding protein (p-CREB) and BDNF.

### 3.3. Effect of Suramin on 6-OHDA-Induced Downregulation of p-ERK, p-CREB, and BDNF Expression

**Figure 3:**
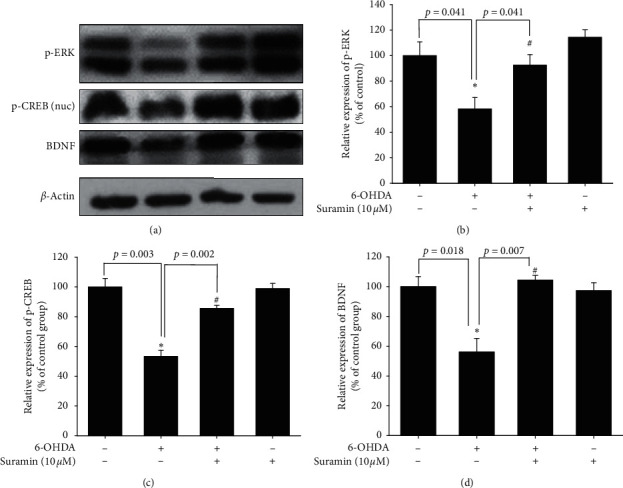
Effect of suramin on 6-OHDA-induced downregulation of phospho-extracellular signal-regulated kinases (p-ERK), phospho-cAMP response element-binding protein (CREB), and brain-derived neurotrophic factor (BDNF) in SH-SY5Y cells. (a) SH-SY5Y cells were pretreated with 10 *μ*M suramin for 1 h and then challenged with 20 *μ*M 6-OHDA for 1 h; western blotting for p-ERK and p-CREB of the control, 6-OHDA, 6-OHDA plus suramin, and suramin alone groups is shown. SH-SY5Y cells were pretreated with 10 *μ*M suramin for 1 h and then challenged with 20 *μ*M 6-OHDA for 8 h; western blotting for BDNF of the control, 6-OHDA, 6-OHDA plus suramin, and suramin alone groups is shown. *β*-Actin was used as an internal control. (b) The quantification results of p-ERK relative density are shown. (c) The quantification results of p-CREB relative density are shown. (d) The quantification results of p-ERK relative density are shown. Data are presented as mean ± SEM, and each value contains three replicates and three samples. ^*∗*^Significantly different from the control group; ^#^significantly different from the 6-OHDA group.

Neuroprotection by BDNF was proven to be mediated through the transient activation of the MAPK pathway and phosphorylation of the CREB downstream pathway. We confirmed the effects of suramin on p-ERK and p-CREB expressions. We found that treatment with 6-OHDA for 1 h significantly decreased p-ERK and p-CREB expressions. However, 10-*μΜ* suramin reversed this 6-OHDA-induced downregulation. Our results demonstrated that pretreatment with 10-*μΜ* suramin for 1 h reversed 6-OHDA-induced downregulation of p-ERK and p-CREB expressions (Figure 3(a), 3(b), 3(c)). We further detected one of the downstream products of the CREB domain, namely, BDNF. Pretreatment with 10-*μΜ* suramin for 8 h significantly reversed 6-OHDA-induced downregulation of BDNF expression. Furthermore, we intend to modulate PTP1B expression to confirm the role of PTP1B in PD(Figure 3(d)).

### 3.4. Effect of PTP1B Knockdown on Neuroprotective Effect on 6-OHDA-Treated SH-SY5Y Cells

**Figure 4:**
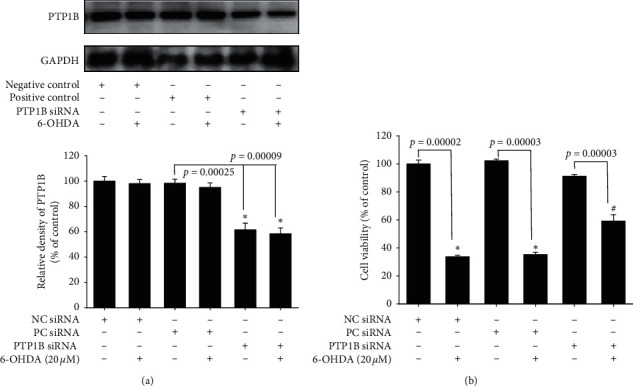
Effect of PTP1B knockdown in SH-SY5Y on PTP1B expression and neuroprotective effect against 6-OHDA damage. (a) SH-SY5Y cells were transfected with siRNAs by LipofectAMINE PlusReagent for 3 h and incubated with new medium overnight. Western blotting for PTP1B of the control (negative control), 6-OHDA (negative control), control (positive control), 6-OHDA (positive control), control (PTP1B siRNA), and 6-OHDA (PTP1B siRNA) groups is shown. Data are presented as mean ± SEM, and each value contains three replicates and three samples. ^*∗*^Significantly different from the control (negative control) group. (b) SH-SY5Y cells were transfected with siRNAs by LipofectAMINE PlusReagent for 3 h and incubated with new medium overnight. Cell viability of the control (negative control), 6-OHDA (negative control), control (positive control), 6-OHDA (positive control), control (PTP1B siRNA), and 6-OHDA (PTP1B siRNA) groups is measured. Data are presented as mean ± SEM, and each value contains three replicates and three samples. ^*∗*^Significantly different from the control (negative control) group. ^#^Significantly different from the 6-OHDA (negative control) group.

First, PTP1B expression with siRNA was knocked down. Twenty-four hours after siRNA transfection, PTP1B protein expression levels were reduced to approximately 58.4%, whereas the positive control (GAPDH) did not affect PTP1B expression in SH-SY5Y (Figure 4(a)). Cell viability data in normal SH-SY5Y showed that treatment with 20 *μ*Μ 6-OHDA significantly reduced the viability from 100% ± 2.7% to 33% ± 1.2% in the negative siRNA group. Moreover, cell viability in the positive siRNA (GAPDH) group after treatment with 20 *μ*Μ 6-OHDA was reduced from 102.1% ± 1.2 to 35% ± 1.6%. Cell viability in the PTP1B knockdown SH-SY5Y was reduced from 91% ± 1.2% to 59% ± 4.6% (Figure 4(b)).

### 3.5. Effect of PTP1B Overexpression on SH-SY5Y Cells against 6-OHDA Damage and Inhibitory Effect on the Neuroprotective Activity of Suramin

**Figure 5:**
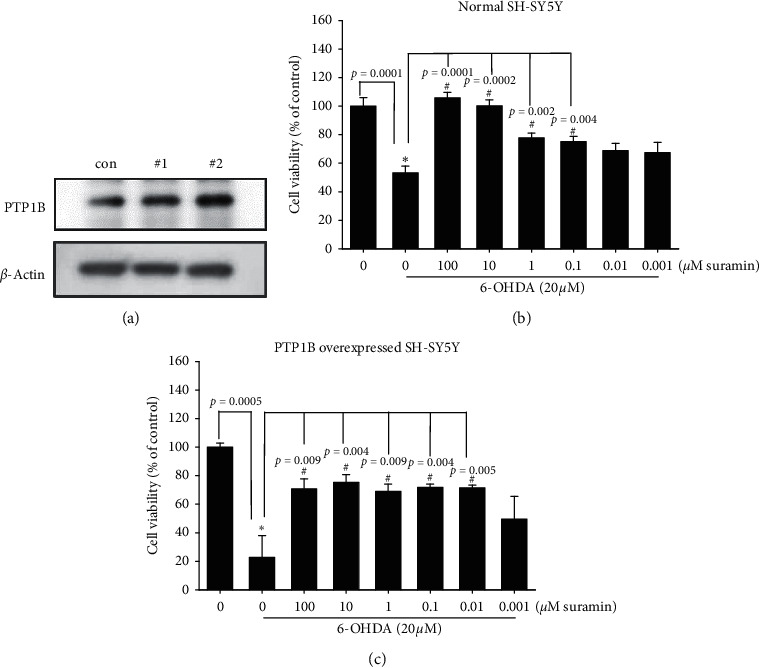
Effect of PTP1B overexpression in SH-SY5Y on PTP1B expression and neuroprotective effect against 6-OHDA damage. (a) PTP1B-overexpressed SH-SY5Y was established by the transfection of PTP1B plasmid. Western blotting for PTP1B of the control, #1 PTP1B-overexpressed SH-SY5Y, and #2 PTP1B-overexpressed SH-SY5Y groups are shown. (b) Normal SH-SY5Y cells were pretreated with 0.01, 0.1, 1, and 10 *μ*M suramin for 1 h and then challenged with 20 *μ*M 6-OHDA for 16 h in the control, 6-OHDA, 6-OHDA plus suramin, and suramin alone groups. Data are presented as mean ± SEM, and each value contains three replicates and six samples. ^*∗*^Significantly different from the 6-OHDA group. (c) PTP1B-overexpressed SH-SY5Y was established by the transfection of PTP1B plasmid. Cell viability of the control; 6-OHDA; and 6-OHDA plus 100, 10, 1, and 0.1 *μ*M suramin groups was measured. Data are presented as mean ± SEM, and each value contains three replicates and three samples. ^*∗*^Significantly different from the control (negative control) group. ^#^Significantly different from the 6-OHDA (negative control) group.

After transfection of the PTP1B-overexpressed vector, stable PTP1B-overexpressed cell clones (#1 and #2) were selected. The PTP1B-overexpressed pattern was confirmed by western blotting (Figure 5(a)). The PTP1B expression was increased from 100% to 132.6% (#1) and 162.9% (#2). The effect of PTP1B overexpression on 6-OHDA damage was performed by assessing cell viability. Treatment with 20 *μΜ* 6-OHDA reduced cell viability from 100% ± 6.0% to 53.3% ± 4.7% in normal SH-SY5Y (Figure 5(b)). However, the PTP1B-overexpressed SH-SY5Y (#2) was treated with 20 *μΜ* 6-OHDA, and cell viability was measured. Our data showed that treatment with 20 *μΜ* 6-OHDA reduced cell viability from 100.0% ± 2.8% to 22.8% ± 15.3% (Figure 5(c)). This result revealed that PTP1B overexpression made SH-SY5Y cells susceptible to damage by 6-OHDA treatment. Moreover, the neuroprotective effect of suramin showed no difference between these two types of SH-SY5Y cells. The pretreatment of 100 *μ*Μ suramin rescued cells from 53% ± 4.7% to 105.5% ± 3.9% and 22.8% ± 15.3% to 70.7% ± 7.0% in normal and PTP1B-overexpressed SH-SY5Y cells, respectively. These two types of cells in the 100 *μ*Μ suramin group rescued cells to maintain almost 50% of cell viability. We then confirmed the inhibitory effect of PTP1B in the zebrafish PD model.

### 3.6. Effect of PTP1B Inhibition on 6-OHDA-Induced Modulation of PTP1B, BDNF, and iNOS mRNA Expression and p-eIF2 and GRP-78 Protein Expression

**Figure 6:**
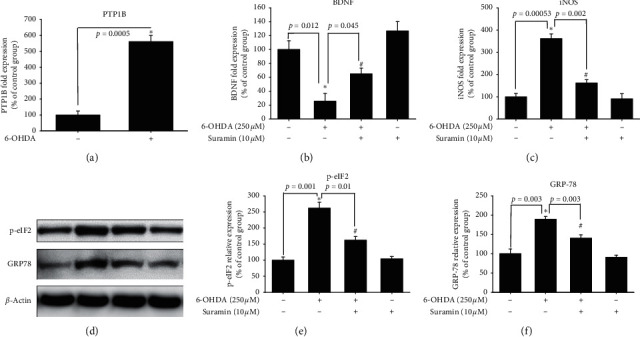
Neuroprotective effect of suramin on the 6-OHDA-induced zebrafish PD model. (a) Zebrafish were pretreated with 10 *μ*M suramin from 9 h after fertilization (hpf) to 3 days after fertilization (dpf) and then challenged with 250 *µ*M 6-OHDA from 2 to 3 dpf. Quantitative PCR of PTP1B for the control and 6-OHDA groups was performed. (b) Zebrafish were pretreated with 10 *μ*M suramin from 9 hpf to 3 dpf and then challenged with 250 *µ*M 6-OHDA from 2 to 3 dpf. Quantitative PCR of BDNF for the control, 6-OHDA, 6-OHDA plus suramin, and suramin alone groups was performed. (c) Zebrafish were pretreated with 10 *μ*M suramin from 9 hpf to 3 dpf and then challenged with 250 *µ*M 6-OHDA from 2 to 3 dpf. Quantitative PCR of iNOS for the control, 6-OHDA, 6-OHDA plus suramin, and suramin alone groups was performed. (d) Zebrafish were pretreated with 10 *μ*M suramin from 9 hpf to 5 dpf and then challenged with 250 *µ*M 6-OHDA from 2 to 5 dpf. Western blotting of p-eIF2 and GRP-78 for the control, 6-OHDA, 6-OHDA plus suramin, and suramin alone groups was performed. (e) Quantitative results of p-eIF2 protein expression. (f) Quantitative result of GRP-78 protein expression. Data are presented as mean ± SEM, and each value contains three replicates and three samples. ^*∗*^Significantly different from the control; ^#^significantly different from the 6-OHDA group.

We then checked neuroprotection, inflammation, and ER stress-related mRNA and protein expression to confirm PTP1B's role in PD. Zebrafish were treated with 10 *μ*M suramin (from 9 hpf to 3 dpf) in the absence or presence of 250 *μ*M 6-OHDA (from 2 to 3 dpf). Quantitative PCR of PTP1B, BDNF, and iNOS was performed with 3 dpf zebrafish. Each sample contained the head tissue of 20 zebrafish larvae. Our data demonstrated that treatment with 250 *μ*M 6-OHDA significantly increased PTP1B mRNA expression in 3 dpf zebrafish (Figure 6(a)). Moreover, we confirmed the expression of BDNF and iNOS in the zebrafish PD model. The potent neuroprotective and neuroregenerative effects of BDNF were proven in PD. Our data showed that treatment with 250 *μ*M 6-OHDA decreased BDNF mRNA expression. However, the pretreatment of 10 *μ*M suramin significantly reversed 6-OHDA-induced downregulation of BDNF (Figure 6(b)), and 10 *μ*M suramin alone slightly increased BDNF expression. The previous study indicated that PTP1B played a vital role in the inflammatory process. Thus, we confirmed iNOS expression. Our results showed that 10 *μ*M suramin significantly attenuated 6-OHDA-induced upregulation of iNOS mRNA expression (Figure 6(c)). Because of the inseparable relationship between PTP1B and ER stress, we then examined ER stress-related proteins, namely, p-eIF2 and GRP-78. Zebrafish were treated with 10 *μ*M suramin (from 9 hpf to 5 dpf) in the absence or presence of 250 *μ*M 6-OHDA (from 2 to 5 dpf). Western blotting of p-eIF2 and GRP-78 was performed using 5 dpf zebrafish. Each sample contained the head tissue of 20 zebrafish larvae. Our data showed that treatment with 250 *μ*M 6-OHDA could increase p-eIF2 and GRP-78 protein expression (Figure 6(d)). However, the pretreatment of 10 *μ*M suramin significantly attenuated 6-OHDA-induced upregulation of p-eIF2 and GRP-78. The quantitative results also showed this trend (Figures 6(e) and 6(f)).

### 3.7. Neuroprotective Effect of Suramin on Zebrafish Locomotor Deficit and Inflammatory-Related mRNA and Protein Expression

**Figure 7:**
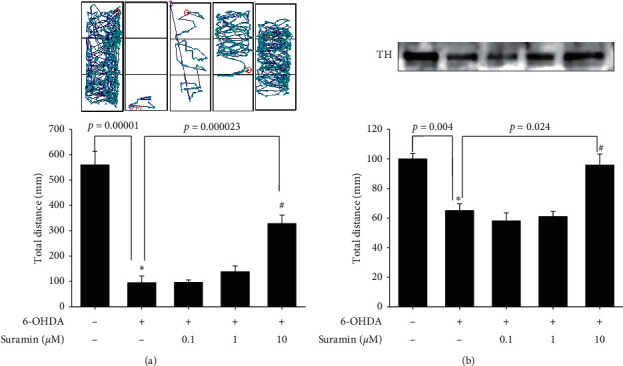
Effect of suramin on 6-OHDA-induced locomotor deficit and tyrosine hydroxylase (TH) expression in the zebrafish PD model. (a) Zebrafish were pretreated with 0.1, 1, and 10 *μ*M suramin from 9 hpf to 5 dpf and then challenged with 250 µM 6-OHDA from 2 to 5 dpf. The upper panel demonstrates a representative swimming pattern, and the lower panel shows the average total swimming distance. (b) Zebrafish were pretreated with 10 *μ*M suramin from 9 hpf to 5 dpf and then challenged with 250 µM 6-OHDA from 2 to 5 dpf. Western blotting of TH for the control, 6-OHDA, 6-OHDA plus suramin, and suramin alone groups was performed. Data are presented as mean ± SEM, and each value represents the mean of 16 samples. ^*∗*^Significantly compared with the control group; ^#^compared with the 6-OHDA group.

**Figure 8:**
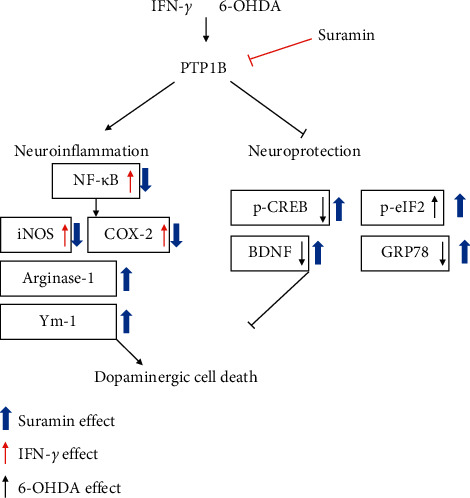
Schematic diagram of PTP1B in IFN-*γ*-induced neuroinflammation and 6-OHDA-induced neuronal death. IFN-*γ* could increase PTP1B expression and further modulate downstream cascade, including the upregulation of proinflammatory cytokines, such as iNOS, COX-2, and NF-*κ*B. Treatment of 6-OHDA could also affect PTP1B and its downstream neuroprotection-related pathway, including the downregulation of p-CREB, GRP-78, and BDNF and upregulation of p-eIF2. Our study revealed that the PTP1B inhibition by suramin could reverse IFN-*γ*-induced upregulation of iNOS and COX-2 protein expression. Moreover, suramin could modulate M2 type microglia-related protein and increase arginase-1 and Ym-1 protein expression. PTP1B inhibition also reversed the 6-OHDA-induced downregulation of p-CREB and BDNF protein expression. In the anti-ER stress section, suramin further enhanced 6-OHDA-induced upregulation of p-eIF2 expression and reversed 6-OHDA-induced downregulation of GRP-78 expression. The effect of suramin significantly protected dopamine neurons against damage.

We finally confirmed the locomotor activity and behavior of zebrafish in the zebrafish PD model. Zebrafish were treated with 0.1, 1, and 10 *μ*M suramin (from 9 hpf to 5 dpf) and in the absence or presence of 250 *μ*M 6-OHDA (from 2 to 5 dpf). Analysis of zebrafish locomotor activity was performed at 5 dpf. The data demonstrated that treatment with 6-OHDA decreased the total swimming distance of zebrafish. Then, the 0.1 and 1 *μ*M suramin plus 6-OHDA groups showed no difference with the 6-OHDA group in the total swimming distance. However, 10 *μ*M suramin significantly reversed the 6-OHDA-induced downregulation of the total swimming distance (Figure 7). We developed a putative diagram to describe the inhibitory effect on PTP1B in PD (Figure 8). IFN-*γ* or 6-OHDA activates microglial inflammatory processes, such as the upregulation of iNOS and COX-2. In neurons, 6-OHDA could inhibit neuroprotection-related proteins p-CREB and BDNF and increase ER stress-related proteins p-eIF2 and GRP-78. PTP1B inhibition by suramin could ameliorate 6-OHDA-induced inflammatory cytokine upregulation, including iNOS and COX-2. Moreover, suramin reversed 6-OHDA-induced downregulation of the expression of the neuroprotective proteins p-CREB and BDNF and ER stress-related proteins p-eIF2 and GRP-78. The data on cell viability and zebrafish locomotor activity also revealed the same trend.

## 4. Discussion

### 4.1. Summary of Our Findings

To date, research on the different ways to modulate inflammation still has its place in drug development [46–48]. However, most studies have investigated the inhibition of specific downstream proinflammatory cytokines, including iNOS, COX-1, and COX-2 [49–51]. Rare studies focused on upstream inflammatory-related targets, including PTP1B, especially in PD. Our studies aimed at PTP1B activity modulation in BV2 murine microglia and SH-SY5Y neuroblastoma cells and zebrafish to expand the potential therapeutic targets and explore PTP1B's role in PD. First, we linked the anti-ER stress activity and antineuroinflammation with PTP1B inhibition, which contributed to the neuroprotective activity. We examined the PTP1B inhibition by suramin that led to the attenuation of IFN-*γ*-induced upregulation of iNOS, COX-2, and NF-*κ*B and increase in the M2 type biomarkers of microglia, such as Ym-1 and arg-1 (Figure 1). No study investigated the effect of modulating PTP1B in neuroinflammation. We also evaluated the antiapoptotic activity of suramin on SH-SY5Y cells. The protection was assessed by alamarBlue. A previous study reported that the TrkB receptor is a direct PTP1B substrate and implicated PTP1B in central BDNF signaling regulation. PTP1B interacts with the activated TrkB receptor in the mouse brain and human SH-SY5Y neuroblastoma cells [3] consistent with our data. We inhibited the PTP1B expression with suramin. This specific inhibition of PTP1B was already demonstrated in early studies [41, 52]. In addition, we confirmed its antiapoptotic activity with the TUNEL assay and caspase-3 protein expression (Figure 2). We also explored the neuroprotective pathways, CREB, and BDNF. Our data showed that the treatment of suramin reversed 6-OHDA-induced the downregulation of p-CREB in nuclear and BDNF protein expression (Figure 3). Furthermore, we reconfirmed the role of PTP1B in neurons with knockdown and overexpressed PTP1B. The knockdown of PTP1B with siRNA protected SH-SY5Y cells from 6-OHDA damage (Figure 4). In contrast, the PTP1B-overexpressed SH-SY5Y cells were susceptible to 6-OHDA toxicity and gained lower cell viability (Figure 5). We used the *in vivo* zebrafish PD model to examine the role of PTP1B. The suramin concentration determined by the survival rate from 0.1 to 10 *μ*M was 100% in 5 dpf (data not shown), and the locomotor activity test demonstrated that 10 *μ*M suramin significantly reversed 6-OHDA-induced downregulation of the total swimming distance and tyrosine hydroxylase (TH) expression (Figure 7). Some inflammation, ER stress, and neuroprotection biomarkers were tested. The data showed that PTP1B inhibition attenuated 6-OHDA-induced upregulation of iNOS, p-eIF2, and GRP-78 and reversed 6-OHDA-induced downregulation of BDNF expression (Figure 6). The relationship between PTP1B and inflammation in the peripheral system was widely investigated more than its protective role.

### 4.2. The Role of PTP1B in the Polarization of Microglia

Several studies demonstrated that the neuroinflammation process contributes to PD progression [53–56]. Considering the damage caused by uncontrolled inflammation to the immune system, microglial polarization is one of the most effective mechanisms that enable the host to maintain the proper immune cell function [57]. Previous studies also showed that PTP1B is involved in inflammation [58–60]. Several studies indicated that the inflammation process is alleviated through PTP1B inhibition by pharmacological intervention and a better pathological process outcome. Much evidence depicted that these effects were related to microglial polarization. Quang et al. showed that the PTP1B inhibitor SF-6013 could significantly attenuate LPS-induced activation of murine macrophage RAW264.7 [61]. Daveri et al. also reported that cyanidin and delphinidin modulated the inflammation process through the attenuation of PTP1B and downregulation of NF-*κ*B [62]. Many studies also discussed the attenuation of inflammation may benefit PD progression in *in vitro* and *in vivo* models [63–67]. Moreover, Xu et al. demonstrated that punicalagin could promote M2 polarization of the macrophage by PTP1B blocking [68]. The same research team also further concluded that the PTP1B inhibition contributes to M2 macrophage polarization, which is involved in reducing microRNA-26a and enhancing MKP1 expression in murine macrophages [59]. The studies mentioned earlier clearly demonstrated the relationship between macrophage polarization and PTP1B. However, the effect of PTP1B modulation on microglia remains ambiguous. Despite this, we inhibited the activity of PTP1B with suramin in murine microglia and showed a similar trend as in the macrophage. PTP1B inhibition led to the attenuation of LPS-induced upregulation of iNOS and COX-2 protein expression and an increase in M2 type biomarker arg-1 and Ym-1 protein expression. Except for the neuroinflammation process, the apoptosis cascade was also involved.

### 4.3. PTP1B Inhibition Is Involved in the Antiapoptotic Pathway

PTP1B was initially considered a tumor suppressor gene. Wiener et al. (1994) first showed that PTP1B expression was significantly increased in the tissue of ovarian cancer patients, which also suggested that PTP1B may play a role in cancer cell proliferation. In their results, 43 of 54 tumors from patients significantly showed upregulation of PTP1B expression associated with C-rebB-2, EGFR, and MCSFR [69]. In a previous study, PTP1B was proved to affect apoptosis via ERK activation and diminished Smad2/Smad3 phosphorylation [70]. Our data showed that treatment with 6-OHDA could increase TUNEL signal, cleave caspase-3, and decrease p-ERK, p-CREB, and BDNF protein expression. Pretreatment with suramin significantly decreased 6-OHDA-induced upregulation of several TUNEL signals; cleaved caspase-3; and downregulation of p-ERK, p-CREB, and BDNF protein expression. Our data showed a similar trend in the recovery effect of neuroprotection-related proteins. PTP1B has been reported to be involved in the ER stress pathway.

### 4.4. The Relationship between ER Stress and Apoptosis in PD

Previous studies showed that telmisartan rescued the rotenone-induced catalepsy symptom in rat PD reversed rotenone-induced lesions of dopamine neurons and inhibited ER stress biomarker, GRP-78, and caspase-12 mRNA [71]. Moreover, basic fibroblast growth factor (bFGF), a member of the FGF family, could rescue neuron death and inhibit GRP-78, CHOP, and caspase-12 in the rat SCI model [72]. Another study indicated that bFGF could improve locomotor deficits; rescue dopamine neuron death; and inhibit GRP-78, CHOP, caspase-12, and XBP-1 protein expression in the 6-OHDA-induced rat PD model [73]. The two studies mentioned earlier also showed that the ER stress biomarker's inhibitory effect was also accompanied by the recovery of dopamine neurons. Our results also demonstrated that the treatment of 6-OHDA significantly increased p-eIF2 and GRP-78 mRNA and protein expression in the zebrafish PD model. PTP1B inhibition by 10 *μ*M suramin significantly attenuated 6-OHDA-induced upregulation and p-eIF2 and GRP-78 expression. The data showed a similar trend with the results mentioned earlier. The improvement effect in dopamine neurons is also the same.

### 4.5. PTP1B-Related Research in the Zebrafish Model

Only a few studies have investigated the role of PTP1B in zebrafish. Van der Sar et al. (1999) first reported that the overexpression of PTP1B in zebrafish produced multiaspect phenotypes, including some lethal effects such as defects of gastrulation and metamere formation. These two effects were induced by the receptor protein tyrosine kinase dephosphorylation [74]. Moreover, the PTP1B inhibitor MSI-1436 demonstrated therapeutic effects in both zebrafish and mouse regeneration models. In the zebrafish, the authors created the injury by amputating 50% of the caudal fin's length. MSI-1436 enhanced cell regeneration rate and proliferation two to three times. Abnormal growth or organ dysfunction was not observed in the recovery section in the MSI-1436 high- or low-dose groups [75]. Furthermore, trodusquemine (MSI-1436) showed no severe side effects in clinical phase I trials. The results mentioned earlier suggested the feasibility of treating PTP1B as a target. Our study first examined the neuroprotective activity of manipulating PTP1B in the zebrafish model, which also showed a similar effect as in the previous research on safety.

## 5. Conclusions

We first showed that PTP1B's inhibition of neuron could improve cell apoptosis. Besides, it could significantly attenuate the interferon-gamma-induced upregulation of proinflammatory cytokines, including iNOS and COX-2. In addition, PTP1B could enhance M2 type microglia markers, such as arginase-1 and Ym-1, in BV2 murine microglial cells. PTP1B inhibition also reversed 6-OHDA-induced downregulation of p-CREB and BDNF in SH-SY5Y cells. On the other hand, we knocked down and overexpressed PTP1B in the SH-SY5Y cell model to recheck its role in neuroprotection. We also verified the role of PTP1B in the zebrafish PD model. Treatment with suramin could significantly reverse the 6-OHDA-induced locomotor deficit and affect some anti-ER stress biomarkers. These results support that PTP1B could potentially regulate PD via antineuroinflammation and antiapoptotic pathways.

## Data Availability

The raw data supporting the conclusions of this manuscript will be made available by the authors, without undue reservation, to any qualified researcher.
